# Exosomal miR-302b as a novel strategy for reversing cellular senescence and promoting healthy aging

**DOI:** 10.20517/evcna.2025.24

**Published:** 2025-04-02

**Authors:** Consuelo Borrás

**Affiliations:** MiniAging Research Group, Department of Physiology, Faculty of Medicine, University of Valencia, CIBERFES, Fundación Investigación Hospital Clínico Universitario/INCLIVA, Valencia 46010, Spain.

**Keywords:** Senescence reversal, miR-302b therapy, aging regeneration

## Abstract

The study by Bi *et al*. (2025) presents “Senoreverse”, an innovative strategy that utilizes exosomal miR-302b to restore the proliferative capacity of senescent cells (SnCs), thus extending lifespan and enhancing cognitive and physical function in aging mice without the risk of tumorigenicity.

Aging is characterized by the progressive decline of physiological functions, partly driven by the accumulation of senescent cells (SnCs) that experience stable cell cycle arrest^[1]^. Cellular senescence has long been viewed as an irreversible process contributing to age-related dysfunctions and diseases. However, recent research by Bi *et al*. (2025) in *Cell Metabolism* presents a groundbreaking strategy termed “Senoreverse”, which leverages exosomal miR-302b to rejuvenate aging mice by reversing the proliferative arrest of SnCs^[2]^. This study highlights the remarkable potential of miR-302b in restoring cell proliferation, extending lifespan, and mitigating age-related pathologies, with no observed tumorigenic risks over a 24-month period.

The focus of this study is miR-302b, a microRNA enriched in human embryonic stem cell-derived exosomes (hESC-Exos), which serves as a powerful modulator of cell cycle re-entry. Through Ago2 Clip-seq analysis, researchers identified its ability to specifically target and suppress the expression of two key cell cycle inhibitors, *Cdkn1a* (*p21*) and *Ccng2*, thereby restoring the proliferative capacity of SnCs. Unlike other senolytic approaches, which concentrate on eliminating SnCs^[3]^, or senomorphic strategies^[4]^, which modulate their secretory activity, this strategy actively re-engages SnCs in the regenerative process.

The beneficial effects of exosomal miR-302b extend beyond cellular proliferation, as evidenced by its impact on lifespan and cognitive function in aging mice. Long-term administration of miR-302b-containing exosomes led to increased median and maximum lifespan and significant improvements in physical performance and memory retention. Behavioral tests, such as rotarod and grip strength assessments, confirmed enhanced motor function, while spatial learning evaluations demonstrated improved cognitive abilities. Furthermore, miR-302b suppresses inflammatory cytokines - including IL-1β, IL-6, and TNF-α - suggesting its role in reducing chronic inflammation, a major driver of aging-related degeneration.

The molecular mechanisms underpinning these effects were provided by single-cell RNA sequencing (scRNA-seq). The analysis revealed that miR-302b treatment reshaped the transcriptomic landscape of aging tissues, increasing the proportion of S-phase cells - a key indicator of cell cycle re-entry - while simultaneously downregulating senescence markers *Cdkn1a* (*p21*) and *Ccng2*. Moreover, genes associated with proliferation, such as *Mki67*, *Top2a*, and *Pcna*, were found to be upregulated, supporting the idea that miR-302b reactivates youthful transcriptional programs within aged tissues.

One of the most critical concerns surrounding cell cycle reactivation strategies is the potential for uncontrolled proliferation, which can lead to tumor formation. Notably, this study reported no increase in cancer incidence or abnormal tissue growth in Exos-302b-treated mice over a 24-month observation period. Postmortem analysis further confirmed the absence of elevated tumor burden, indicating that miR-302b selectively promotes physiological proliferation without triggering oncogenic transformation. However, much more work is needed in the future to assess any side effects of long-term use, such as tumorigenesis.

## POTENTIAL IMPACT AND FUTURE DIRECTIONS

**Figure 1:**
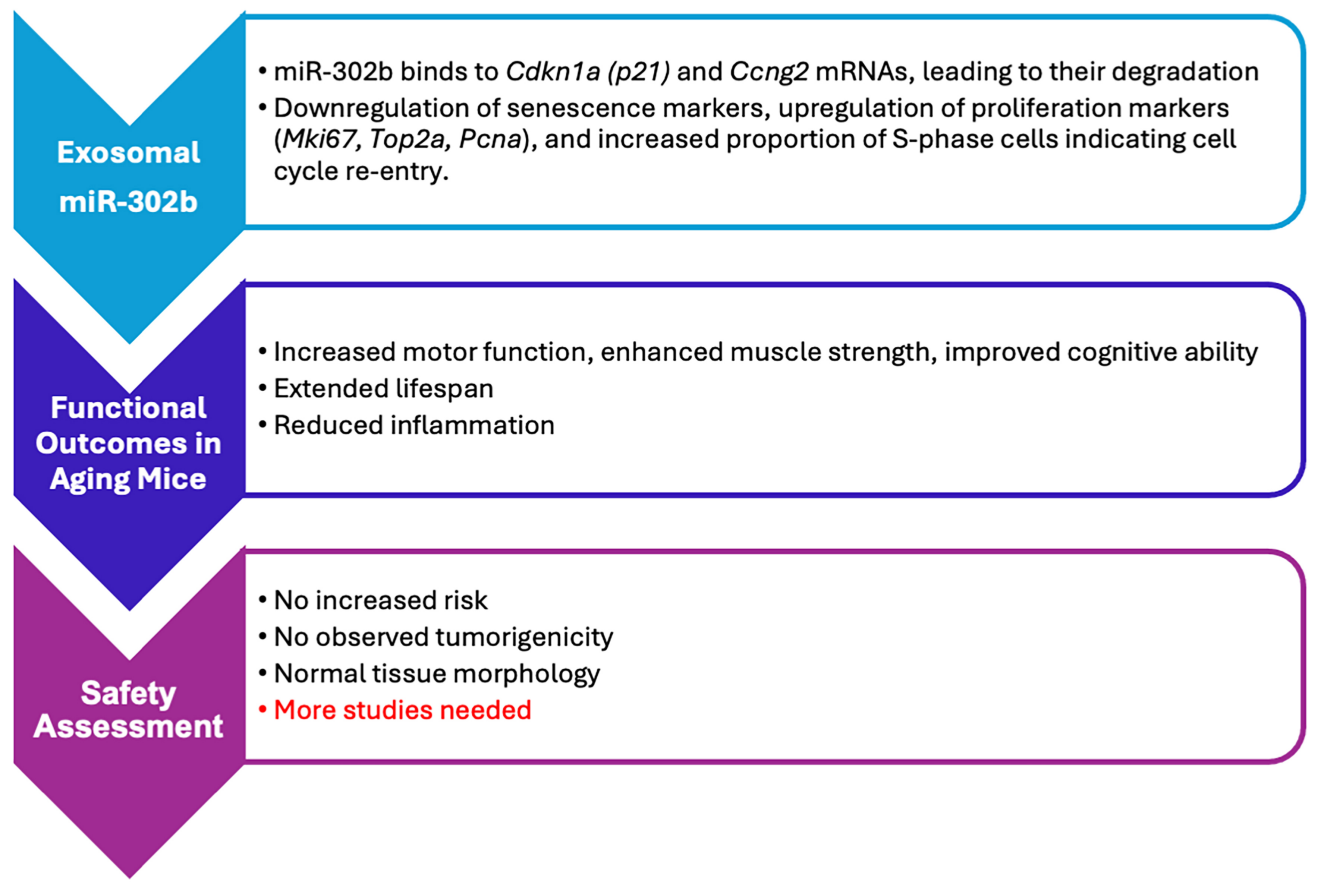
Mechanism and effects of exosomal miR-302b in senescence reversal and healthy aging.

These findings signify a paradigm shift in aging research, challenging the long-held belief that senescence is irreversible. By demonstrating that SnCs can be reprogrammed into proliferative cells, this study opens the door to novel rejuvenation therapies focusing on restoring cellular function rather than eliminating SnCs [Figure 1].

The implications of miR-302b therapy extend beyond the process of aging itself, with potential applications in treating various age-related diseases. Given its ability to reduce inflammation, enhance cognition, and restore tissue homeostasis, miR-302b could serve as a therapeutic candidate for conditions such as neurodegenerative disorders (e.g., Alzheimer’s and Parkinson’s disease), osteoarthritis, osteoporosis, cardiovascular diseases, and metabolic syndromes.

Despite these promising results, several hurdles must be overcome before clinical translation. Optimizing delivery methods remains a priority, as exosome-based systems enhance cellular uptake but require refinement for human applications. Additionally, rigorous human trials must validate long-term safety and efficacy, ensuring that miR-302b does not induce tumorigenesis or introduce other unforeseen risks. Regulatory approval will also be critical, necessitating standardized production and quality control measures to facilitate widespread therapeutic use.

## CONCLUSION

Bi *et al*. (2025) provide compelling evidence that exosomal miR-302b can effectively rejuvenate aging mice by reversing SnC-induced proliferative arrest^[2]^. By directly targeting *Cdkn1a* and *Ccng2*, this approach offers a novel strategy for systemic rejuvenation with significant implications for human longevity and healthspan. If validated for long-term safety and efficacy and successfully translated to clinical settings, miR-302b-based therapies could revolutionize the field of regenerative medicine, providing a viable intervention for age-related diseases and enhancing the quality of life in aging populations.

## DECLARATIONS

### Copyright

© The Author(s) 2025.
